# Facilitators for developing an interprofessional learning culture in nursing homes: a scoping review

**DOI:** 10.1186/s12913-023-09092-5

**Published:** 2023-02-21

**Authors:** Frank H. O. Verbeek, Merel E. A. van Lierop, Judith M. M. Meijers, Erik van Rossum, Sandra M. G. Zwakhalen, Miranda G. H. Laurant, Anneke J. A. H. van Vught

**Affiliations:** 1 HAN University of Applied Sciences, School of Health Studies, Research Group Organisation of Healthcare and Services, Nijmegen, The Netherlands; 2 Maastricht University, Care and Public Health Research Institute, Department of Health Services, Maastricht, The Netherlands; 3 Living Lab in Ageing and Long-Term Care, Maastricht, The Netherlands; 4 Zuyderland Care, Zuyderland Medical Center, Sittard-Geleen, the Netherlands; 5 Zuyd University of Applied Sciences, Research Centre for Community Care, Academy of Nursing, Heerlen, The Netherlands

**Keywords:** Interprofessional learning, Learning culture, Nursing home, Interprofessional relations (MESH), Quality of health care (MESH)

## Abstract

**Background:**

Healthcare professionals in nursing homes face complex care demands and nursing staff shortages. As a result, nursing homes are transforming into home-like personalised facilities that deliver person-centred care. These challenges and changes require an interprofessional learning culture in nursing homes, but there is little understanding of the facilitators that contribute to developing such a culture. This scoping review aims to identify those facilitators.

**Methods:**

A scoping review was performed in accordance with the *JBI Manual for Evidence Synthesis* (2020). The search was carried out in 2020–2021 in seven international databases (PubMed, Cochrane Library, CINAHL, Medline, Embase, PsycINFO and Web of Science). Two researchers independently extracted reported facilitators that contribute to an interprofessional learning culture in nursing homes. Then the researchers inductively clustered the extracted facilitators into categories.

**Results:**

In total, 5,747 studies were identified. After removing duplicates and screening titles, abstracts and full texts, 13 studies that matched the inclusion criteria were included in this scoping review. We identified 40 facilitators and clustered them into eight categories: (1) shared language, (2) shared goals, (3) clear tasks and responsibilities, (4) learning and sharing knowledge, (5) work approaches, (6) facilitating and supporting change and creativity by the frontline manager, (7) an open attitude, and (8) a safe, respectful and transparent environment.

**Conclusion:**

We found facilitators that could be used to discuss the current interprofessional learning culture in nursing homes and identify where improvements are required. Further research is needed to discover how to operationalise facilitators that develop an interprofessional learning culture in nursing homes and to gain insights into what works, for whom, to what extent and in what context.

## Introduction

Healthcare professionals in nursing homes have to deal with increasingly complex care demands and nursing staff shortages [1, 2]. In addition, nursing homes are transforming from medical-oriented institutional settings to more home-like personalised facilities. This leads to more patient-centred care that considers the residents’ preferences and needs and integrates innovations and new technology into daily practice [3]. These changes require healthcare professionals to have specific expertise, flexibility, adaptability and the ability to work and learn more intensively together in daily practice [4, 5].

Insights from interprofessional collaborative behaviour frameworks and continuous learning practices are important to developing an interprofessional learning culture [6–9]. Such a culture requires an environment in which at least two healthcare professionals work and learn together to provide the best quality of care to nursing homes residents [7–9]. Methods for developing an interprofessional learning culture have been studied more often in hospitals, primary care and in education [10, 11]. For example, to stimulate collaboration within interprofessional teams, the Interprofessional Education Collaborative identified four core competency domains: values and ethics, roles and responsibilities, communication and teamwork, and team-based care to improve health outcomes [12].

The concept of ‘just-in-time learning’ is especially recommended for developing continuous learning practices in a nursing home setting. With just-in-time learning, learning takes place anywhere, anytime and anyhow using real-time complex cases in daily practice [13]. This combination of working and learning can also be described as creating a workplace culture in which informal learning takes place in daily practice with the aim of improving employees‘ competencies and leadership, enhancing their knowledge, and improving the quality of care and work [14–16]. However, it is challenging to develop an interprofessional learning culture in nursing homes.

To improve the interprofessional learning culture and person-centered care all professionals in nursing homes have to collaborate intensively together whereas we have to take into account that different settings may emphasise and organise interprofessional collaboration differently [5, 17, 18]. For example, Community Living Centres in the United States use a quality improvement approach called CONCERT to bring together diverse members of the healthcare team. CONCERT includes strategies to learn from the bright spots, observe; collaborate in huddles; and keep it bite-sized [19]. However, professionals from various healthcare professions should be involved in patient-centred care, they are often organised in separate teams and may therefore hardly know each other. Professionals are more often focused on their own tasks and responsibilities and are unaware of the roles or tasks of other professions [20]. An example of this siloed work is the work of nursing teams, mainly consisted of licensed practical nurses and the work of physicians and allied health professionals. Both set their own care goals or treatment goals, separately from each other. This is contrary to person-centred care and underlines the importance of interprofessional collaboration [21]. Moreover, professionals are not yet accustomed to sharing their knowledge and expertise, which can reduce the quality of care for nursing home residents [20].

An interprofessional learning culture in nursing homes must be developed to improve the quality of patient-centred care, fulfil increasingly complex care demands, and deal with staff shortages. However, there has been no overview made of the facilitators that contribute to developing such a culture. The purpose of this scoping review is to outline those facilitators.

## Methods

A scoping review was performed in accordance with the method in the *JBI Manual for Evidence Synthesis* (2020) [22].

### Strategy, search terms and search string

The literature search for this scoping review was carried out from January 2020 to January 2021. The search strategy comprised subsequent steps as proposed in the JBI manual [22]. First, we used the PubMed and CINAHL databases to identify relevant keywords for our search string. Then we used those keywords to build an elaborated search string. A research librarian from HAN University of Applied Sciences and two researchers (FV, MvL) helped to define terminology by searching for synonyms and broadening definitions in the search strategy. The search string was discussed with all authors. The search strategy was improved to increase its sensitivity and reduce the risk of missing relevant studies (Table 1). The search was performed in seven databases: PubMed, Cochrane Library, CINAHL, Medline, Embase, PsycINFO and Web of Science.

**Table 1:** Search terms

Term	Keyword(s)
Interprofessional learning culture^*^	Interprofessional collaboration Interprofessional practice Interprofessional working Integrated collaboration Collaborative practice Learning culture Working culture Workplace learning Just-in-time learning Informal learning Workplace training Workplace-based learning Workplace education
Nursing home^*^	Convalescence home Long-term care Residential care Care home Rehabilitation centre Geriatric ambulatory centre Elderly house

^*^ Interprofessional learning culture: "Patient Care Team"[mesh] OR multidisciplinar*[ti] OR Interdisciplinar*[ti] OR collaborat*[ti] OR interprofessional*[ti] OR working culture*[ti] OR learning culture*[ti] OR Patient Care Team*[ti] OR Medical Care Team*[ti] OR Healthcare Team*[ti] OR Health Care Team*[ti] OR intraprofessional[ti] OR intra professional[ti] OR intra sector*[ti] OR inter sector*[ti] OR Care coordinat* OR intra sector*[ti] OR Integrated care[ti] OR integrated health[ti] OR coordinated care[ti] OR comprehensive care[ti] OR seamless care[ti] OR transmural care[ti]

^*^ Nursing Home: "Nursing Homes"[mesh] OR convalescene home*[tiab] OR long term car*[tiab] or residential car*[tiab] OR nursing home*[tiab] OR care home*[tiab] OR geriatric ambulator*[tiab] OR rehabilitation centre*[tiab] OR rehabilitation center*[tiab] OR elderly hous*[tiab]

### Inclusion criteria

#### Types of participants

The search was limited to the interprofessional team working at a nursing home. An interprofessional team is defined as a team in which at least two healthcare professionals from different professions intensively work and learn together in daily practice to manage residents’ care and share their specialised knowledge, skills or abilities to innovate this care [23, 24].

#### Concept

We were interested in the facilitators that contribute to creating an interprofessional learning culture in nursing homes. Facilitators were defined as any relevant factors, elements or actions.

#### Context

The context for this scoping review includes nursing homes and their working healthcare staff. A nursing home is a public or private residential care home that provides a high level of long-term personal nursing and medical care for older adults and chronically ill patients who cannot care for themselves properly [25].

#### Types of evidence sources

We included quantitative, qualitative, action research and mixed method designs to retrieve findings published in the last five years (2016–2021). Case reports (n = 1 studies) were excluded because of a possible lack of generalisability. We excluded information from books, book chapters and (newspaper) interviews because we were only interested in results from peer-reviewed studies. Studies in English or Dutch were selected.

#### Search strategy

The identified records were imported from ©2021 Rayyan into EndNote X8 for further investigation and selection. The first step in EndNote X8 was to remove all the duplicates in seven steps, based on author, year, title, pages, volume, issue, journal and secondary title. Bramer et al. published a detailed description of these steps [26]. After duplicates were removed, one researcher (FV) independently screened the titles and abstracts of the initial studies based on the inclusion criteria for possible inclusion in this scoping review. Each study was marked ‘inclusion’, ‘exclusion’ or ‘maybe’. Two researchers (FV and MvL) discussed the studies marked ‘inclusion’ or ‘maybe’. For the two studies where no consensus was reached, two independent researchers (AvV, JM) were asked to assess them. After this process, two full text articles were selected randomly and independently analysed by the two authors (FV, MvL) for calibration regarding inclusion. Findings from and (dis)agreements about these two studies were discussed before the other full text articles were analysed by both authors.

### Data extraction and analysis

First, we extracted characteristics from all the included studies. Second, we extracted data about the facilitators for developing an interprofessional learning culture in nursing homes. These facilitators were extracted independently by two researchers (FV, MvL) and placed in a table to create a first overview.

After extraction, the two researchers discussed the similarities and differences in their independent findings. During this discussion, they analysed each finding regarding the facilitators and clustered the findings into categories. The researchers focused on finding categories and reporting these categories until no more new categories were found. After the results were assigned to categories and the two researchers reached agreement, the results were presented to two other researchers for agreement and a final check (AvV, JM). Disagreements were discussed until consensus was reached.

## Results

**Fig. 1:**
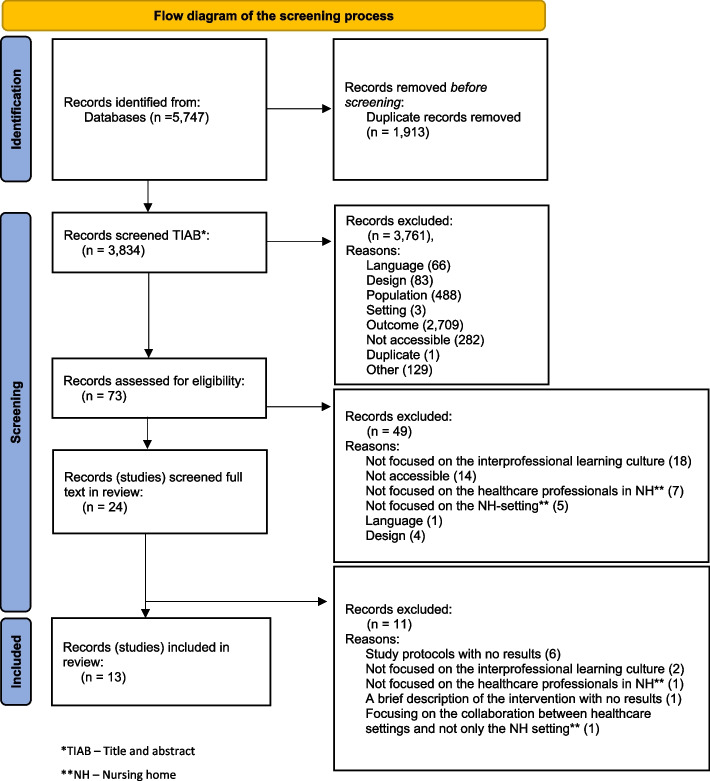
Flow diagram of the screening process

In total, 5,747 studies were found. After removing duplicates, 3,834 studies remained and were screened based on title and abstract. After screening and discussion between the researchers, 73 studies were assessed for eligibility for full-text screening. After this process, 13 studies were included in this scoping review (Fig. 1).

### Characteristics of the included studies

Thirteen studies were included. They originated from different countries and most applied a qualitative design (two studies included action research and one study used a quantitative design; see Table 2).

**Table 2:** Characteristics of the included studies

#	Author, year and country	Design	Population studied	Aim of the study
1	*Anvik *et al*., 2020, Norway* [27]	Qualitative	Healthcare professionals	To investigate the conditions under which learning and innovation occur in nursing homes
2	*Fleischmann *et al*., 2017, Germany* [28]	Qualitative	Healthcare professionals	To explore how nurses experience general practitioners’ visits to the nursing home and interprofessional communication and collaboration
3	*Folkman *et al*., 2019, Norway* [29]	Qualitative	Frontline managers collaborating daily with healthcare professionals	To examine how frontline managers facilitate interprofessional collaboration in three health care services, with a special focus on managing social educators and nurses in their daily practice
4	*Goller *et al*., 2019, Germany* [30]	Qualitative	Nurses and nurse aides	To investigate learning and development processes of newly employed nurse aides
5	*Hurlock-Chorostecki *et al*., 2016, Canada* [31]	Qualitative	Healthcare professionals	To identify, from the healthcare professionals’ perspective, nurse practitioner strategies used to enhance interprofessional care
6	*Khemai *et al*., 2020, The Netherlands* [32]	Quantitative	Healthcare professionals	To examine the perceptions and needs of nurses regarding collaboration with other nurses, other professionals, people with dementia, and loved ones, and to investigate whether these perceptions and needs differ between healthcare settings and among three levels of nursing
7	*Kim *et al*., 2020, South Korea* [33]	Qualitative	Practitioners and professors	To develop a conceptual framework to structure the shared roles and tasks of interdisciplinary teams for efficient function-focused care of nursing home residents
8	*Müller *et al.,* 2018, Germany* [34]	Qualitative	Healthcare professionals	To develop and test measures to improve collaboration and communication between nurses and general practitioners in this setting
9	*O’Leary 2016, United States* [35]	Action research	Healthcare professionals	To outline aspects of an action research study examining the emergence of effective communication, shared decision-making and knowledge sharing within change management teams
10	*Park *et al*., 2019, South Korea* [36]	Qualitative	Healthcare professionals	To clarify the regularity of sharing commonly used information and knowledge across disciplines, and to develop a practical care strategy specialised for nursing homes
11	*Stühlinger *et al*., 2019, Switzerland* [37]	Qualitative	Healthcare professionals in rehabilitation homes	To test the relationship of a shared language in interprofessional healthcare teams
12	*Tsakitzidis., *et al*., 2017, Belgium* [38]	Qualitative	Healthcare professionals	To gain insights into professionals’ perceptions of interprofessional collaboration in nursing homes and the factors that affect interprofessional collaboration
13	*Venturato *et al*., 2019, Australia* [39]	Action research	Healthcare professionals	To address the need for sustainable culture change in residential aged care by developing and piloting a novel workforce development intervention (Towards Organisational Culture Change)

### Categories

We identified 40 facilitators in the 13 studies. These were clustered into eight categories: (1) shared language, (2) shared goals, (3) clear tasks and responsibilities, (4) learning and sharing knowledge, (5) work approaches, (6) facilitating and supporting change and creativity by the frontline manager, (7) an open attitude, and (8) a safe, respectful and transparent environment (Table 3).

**Table 3:** Facilitators for developing an interprofessional learning culture

*Categories *	*Facilitators*
*Shared language*	- Consult with colleagues within your own discipline, in other disciplines and outside your organisation - Use a communication protocol - Improve communication skills regarding residents - Create and develop new relationships - Focus on how to communicate - Focus on a shared language - Use name badges
*Shared goals*	- Create a common vision - Use a framework (e.g., function-focused care)
*Clear tasks and responsibilities*	- Have well-known tasks and responsibilities for all professionals on a team - Use the nurse to communicate to the physician(s) - Have transparent definitions of tasks - Use formal time schedules to discuss each other’s roles - Have clear roles
*Learning and sharing knowledge*	- Deliver training to improve knowledge about how to collaborate interprofessionally - Develop knowledge and associated skills about culture change - Work with Evidence Based Practice (EBP), discuss care rationales and share knowledge - Use a preliminary care model or a change cycle - Use knowledge to identify residents’ issues - Guide the learning activities - Structure learning activities - Improve skills and support
*Work approaches*	- Contextualise the nursing home as a site for learning and innovation - Work with a holistic approach and continuous assessment - Focus on practical information about how to guide people - Use practice-based learning opportunities - Take time to focus on the resident - Use a systematic approach
*The frontline manager facilitating and supporting change and creativity*	- Frontline managers must have innovative solutions - Frontline managers must have clear leadership
*An open attitude*	- Pay attention to social and formal processes - Have an open and flexible way of working - Have a natural attitude and be involved
*A safe, respectful and transparent environment*	- Use a concept to focus on safety (e.g., the Team Psychological Safety Concept) - Have an open and transparent perspective on each other - Appreciate and respect each other - Create an environment in which people feel safe - Listen to each other’s opinions - Negotiate respectfully - Create a safe team climate

#### Shared language

Seven studies reported findings related to having a shared language [28, 29, 31, 32, 34, 36, 37]. Each professional has their own expertise, background and educational level, which often results in different professional languages and phrases being used to describe the same phenomena. This makes it challenging to communicate and coordinate in an interprofessional team.

It is recommended that a shared language be used in the interprofessional teams [28, 29, 36, 37] and with colleagues outside the organisation [32]. Further, using a communication protocol like the Situation, Background, Assessment and Recommendation (SBAR) protocol (28), facilitating communication competencies and paying attention to how a team communicates will improve the interprofessional learning culture [28, 29, 31, 32, 34, 36, 37].

#### Shared goals

Two studies reported that creating shared goals with professionals from different professions is important to an interprofessional learning culture [33, 38]. For example, shared goals could relate to improving quality of care and quality of life for older residents [33]. These goals should be balanced across different professions.

To help establish these shared goals, they could be set through a process mediated by a coordinator. Kim et al. (2020) found that using a theoretical approach can help to translate goals into practice (e.g., the function-focused care approach in nursing homes) [33]. Interprofessional education also may help in developing a common vision and goals related to person-centred care [38].

#### Clear tasks and responsibilities

Three studies reported that it is important to have clear roles, tasks and responsibilities in an interprofessional team that are well-known to all professionals on the team [29, 31, 32]. For example, it can be beneficial to have the nurse on the interprofessional team take a clear role as central communicator with the physician(s) involved [31]. In this role, the nurse is a central point of contact for other professionals or colleagues and could bridge the gap in language, knowledge and skills between professionals on an interprofessional team. It was observed that the holistic point of view of nursing helped the nurse practitioner create clarity in care plans and implement them with all professionals involved [31]. Another study mentioned that it is important to schedule formal meetings to discuss each other’s roles and tasks in daily practice [32].

#### Learning and sharing knowledge

Six studies described facilitators related to learning skills and exchanging knowledge (e.g., improving skills and knowledge about the residents’ diseases and support for how to guide people when working together as one team) [30–32, 37–39]. To improve knowledge sharing on an interprofessional team, team members need to: 1) work with evidence-based practice, and 2) be aware and discuss the care rationales [31].

Furthermore, learning activities need to be structured [30]. This could help team members better understand the learning and development process of (new) colleagues and how to facilitate this development process. Two studies mentioned developing professionals’ knowledge and skills regarding culture change using a change cycle, which can foster an interprofessional learning climate [37, 39]. For example, the QPAR (Question, Plan, Act and Reflect) cycle was mentioned in one article [39]. Professionals confirmed that using a change cycle, such as QPAR, improves the structure in a meeting and improves working together as one team on one specific important subject [39].

Additionally, offering pedagogically rich learning activities together with goal-directed guidance and direct guidance can foster an interprofessional learning climate. An example from one study was having more experienced nurses introduce new tasks to other healthcare professionals [30]. The instructing nurse explains what has to be done and why, and then the experienced nurse models that task. After the healthcare professionals who are being trained observe the task, they perform it by trying to imitate the more experienced nurse. Their performance is assessed by an instructor and feedback is given if necessary.

#### Work approaches

Work approaches that differ from the often-classic approaches used in healthcare are needed to create a profound interprofessional learning culture [27–29, 31–33, 36, 37]. These might include working with a holistic approach and with continuous assessment to stay up to date about a resident’s health status [33]. To work holistically and with continuous assessment, it is recommended that all relevant information be shared among all professionals on a team [29]. Furthermore, frontline managers should ‘use a systematic approach to exploit the opportunity presented by the variety of competence available’ to improve interprofessional working [29].

Nursing homes also must be a place for practice-based learning opportunities. This requires a work approach in which informal and formal learning situations are created with a focus on learning in everyday practice and on contributing practical information to the interprofessional learning culture [27]. For example, nurses need practical information and advice about aligning care agreements between healthcare providers [32].

Further, one study concluded that ‘time to focus on the patient’ contributes to interprofessional care. However, healthcare professionals stated that they did not have this time in daily care [31]. It also can be helpful to involve nurses in general practitioners’ visits to nursing homes. This can prevent delays when there is a sudden need for assistance or information [28]. However, in some cases, a nurse’s attendance can also be seen as undesirable. For example, a confidential atmosphere in a private conversation (without a nurse present) can boost the general practitioner’s relationship with the resident and result in more productive performance [28].

#### Facilitating and supporting change and creativity by the frontline manager

One study showed that managers play an important role in coaching individuals to translate their ideas and beliefs into interprofessional efforts in practice [29]. Managers must be able to facilitate change and support creativity in a setting where many healthcare professionals work together with their own responsibilities, experiences and tasks. Managers have to pay attention to using different competencies, adopting and implementing new approaches and responsibilities, and the division of roles and tasks [29].

#### An open attitude

Two studies reported on the attitudes of healthcare professionals [29, 38]. Ideally, these attitudes should be characterised by equality rather than hierarchy. They also need to be open, holistic and flexible [29] Frontline managers described this open and holistic way of working as more innovative than continuing to emphasise the differences between professionals and fixed responsibilities and duties [29]. It is important to avoid conflicts arising from ideas about formal and social processes in the collaboration [38].

#### A safe, respectful and transparent environment

Five studies mentioned the importance of creating a safe and respectful environment in an interprofessional learning culture in nursing homes [31, 32, 34, 35, 37]. For example, nurses and general practitioners indicate that mutual respect and appreciation of their different professions improve their mutual relationship [34]. Khemai et al. (2020) showed that one of 17 reported needs in interprofessional collaborations was the need to feel safe about implementing care agreements that have been made [32]. Having respectful negotiations was another important factor that influences collaboration [31].

Additionally, a safe team climate was mentioned as an important influencing factor, and the Team Psychological Safety (TPS) concept contributes to a safe team climate [35, 37]. TPS has been defined as ‘an atmosphere within a team where individuals feel comfortable engaging in discussion and reflection without fear of censure’ [35]. This concept includes the possibility for all the professionals on a team to raise issues or problems in daily practice [35]. Finally, there is a need for transparency about diagnosis and therapy, reliable, clear and well-substantiated reports, and a clear clarification of responsibilities and expectations from each other [31, 34].

## Discussion

In this scoping review, we identified 40 facilitators clustered in eight categories: (1) shared language, (2) shared goals, (3) clear tasks and responsibilities, (4) learning and sharing knowledge, (5) work approaches, (6) facilitating and supporting change and creativity by the frontline manager, (7) an open attitude, and (8) a safe, respectful and transparent environment. These categories form a basis for developing and improving an interprofessional learning culture in nursing homes.

Several categories specific to the nursing home setting correspond to elements of interprofessional educational and competency frameworks in other healthcare settings. For example, the Canadian Interprofessional Health Collaborative Framework (2010) states that three categories are essential to an interprofessional learning culture: 1) communication in a team, 2) clear roles, tasks and responsibilities and 3) using each other’s knowledge [40]. Furthermore, three best practice models of interprofessional education for healthcare professionals, focusing on healthcare students as future interprofessional team members, report similar categories such as responsibility, coordination, communication, trust, respect and sharing knowledge with each other [41].

When zooming in on the nursing home setting, there was more emphasis on facilitators about having a shared language, having a safe respectful and transparent environment, and stimulating learning and sharing knowledge. The greater attention to these facilitators can be explained by the challenges of daily care in nursing homes. We discuss three explanations.

First, many nursing homes only provide room and board care to residents who are aided by minimally trained or untrained staff and receive little or no input from physicians or nurses [42]. As the complexity of the demand for nursing care increases, more well-trained certified nurse assistants (CNA), nurses and professionals from other professions (including medical and allied healthcare professionals) should be added to the skill mix to maintain high-quality care. For example, a study in the US shows that adding well-trained CNA's (with increased requirements for CNA training) are able to improve the quality of long-term care [43]. However, adding well-trained professionals to a team is challenging. Great variety in education levels could hinder the use of each other’s knowledge and expertise (e.g. because each professional speaks their own professional language used in their own field or within their own education level) [18]. It is crucial to pay attention to the mix of education levels and different views on good quality of care.

Second, the way nursing homes are organised influences collaborating and learning within an interprofessional team. The different settings may emphasise and organise interprofessional collaboration differently. For example, nursing home staff in the Netherlands and England work closely with other medical healthcare professionals (such as physicians) and could form a team. In other countries, for example in Germany, nursing homes mainly employ nursing staff/assistants. The nurses could consult the physician, but there is no frequent or daily collaboration with a general physician [44]. In that case, professionals from other professions are available remotely from other organisations [42]. This may make it difficult for various professionals to learn together and share knowledge because they do not commonly work intensively together, and it could be more difficult to understand each other’s daily work. From the organisational perspective, it is important to facilitate interprofessional learning (e.g., by contextualising the nursing home as a site for learning and innovation, or working with a holistic approach and continuous assessment) to improve quality of care or to use systematic approaches to work together [27, 29].

Finally, current daily practices could explain the attention paid to learning and sharing knowledge. Nursing homes increasingly face challenges in delivering complex, home-like, person-centred care with limited staff. Making time for interprofessional learning is not usually part of the culture in nursing homes, nor is critically reflective behaviour by professionals [3, 20]. Thus, there is still a culture of name, blame and shame in many nursing homes [45]. Such an atmosphere could hinder professionals from communicating openly or sharing insecurities or mistakes. Culture change is difficult and takes time.

The 40 facilitators found in this review can contribute to developing and strengthening an interprofessional learning culture in nursing homes.

### Limitations

Although we found 13 studies including 40 facilitators for developing an interprofessional learning culture in nursing homes, the operationalisations of the facilitators described in the extracted studies were limited. Therefore, the meaning of a specific facilitator was not always clear. For example, the studies mentioned the importance of focusing on tasks and responsibilities, but they included no detailed description of how or with which specific methods and for whom to do that. Thereby, we included studies with facilitators that contribute to the development of an interprofessional learning culture. It is possible that we missed relevant studies due this inclusion criteria. Some studies operationalise facilitators regarding of quality improvements in collaboration instead of interprofessional learning cultures. For example, in the Hartmann et al. study where same facilitators were mentioned to improve quality of care, communication, collaboration and positive work experiences which are also important elements for an interprofessional learning culture in nursing homes [19].

### Recommendations

Further research should focus on operationalising the facilitators in more detail and explaining how they contribute to an interprofessional learning culture in nursing homes. This should include more detail about the preconditions and results on patient, professional and organisational levels. We need to create more understanding about what works, for whom, to what extent and in what context.

This information would make it possible to build and evaluate a practical guide about how to develop an interprofessional learning culture in nursing homes. Such a guide could help people evaluate a situation with regard to the facilitators or categories, and help them assess where improvements need to be made in a nursing home’s interprofessional learning culture. It is important to look at an organisation’s specific context and tailor the facilitators to it. This tailoring should be bottom-up in consultation and co-creation with the entire interprofessional team. Doing it in this way will make healthcare professionals more motivated to work on establishing an interprofessional learning culture [36].

## Conclusion

This scoping review identified eight categories of facilitators that can support the development of an interprofessional learning culture in nursing homes. These categories include (1) shared language, (2) shared goals, (3) clear tasks and responsibilities, (4) learning and sharing knowledge, (5) work approaches, (6) facilitating and supporting change and creativity by the frontline manager, (7) an open attitude, and (8) a safe, respectful and transparent environment. Further research is needed to operationalise these facilitators in more detail so we can gain insights into what works, for whom, to what extent and in what context.

## Data Availability

The datasets generated and/or analysed during the current study are available through the corresponding author on reasonable request.
